# A Community-Based Intervention to Enhance Subjective Well-Being in Older Adults: Study Design and Baseline Participant Profiles

**DOI:** 10.3390/healthcare12030322

**Published:** 2024-01-26

**Authors:** Tsubasa Nakada, Takako Kozawa, Satoshi Seino, Shinichi Murota, Miki Eto, Junko Shimasawa, Yumiko Shimizu, Shinobu Tsurugano, Fuminori Katsukawa, Kazunori Sakamoto, Hironori Washizaki, Yo Ishigaki, Maki Sakamoto, Keiki Takadama, Keiji Yanai, Osamu Matsuo, Chiyoko Kameue, Hitomi Suzuki, Kayo Kurotani, Kazunori Ohkawara

**Affiliations:** 1 Graduate School of Informatics and Engineering, The University of Electro-Communications, Tokyo 182-8585, Japan; 2 Faculty of Human Health, Komazawa Women’s University, Tokyo 206-8511, Japan; 3 Research Team for Social Participation and Healthy Aging, Tokyo Metropolitan Institute for Geriatrics and Gerontology, Tokyo 173-0015, Japan; 4 Faculty of Humanities and Social Sciences, Tokyo Metropolitan University, Tokyo 192-0397, Japan; 5 Faculty of Human Sciences, Osaka University of Economics, Osaka 533-8533, Japan; 6 School of Nursing, The Jikei University, Tokyo 182-8570, Japan; 7 Center for Health Sciences and Counseling, Kyushu University, Fukuoka 819-0395, Japan; 8 Sports Medicine Research Center, Keio University, Yokohama 223-8521, Japan; 9 Green Computing Systems Research Organization, Waseda University, Tokyo 169-8050, Japan; 10 Faculty of Science and Engineering, School of Fundamental Science and Engineering, Waseda University, Tokyo 169-8050, Japan; 11 Research Center for Realizing Sustainable Societies, The University of Electro-Communications, Tokyo 182-8585, Japan; 12 Office for Research Strategy, The University of Electro-Communications, Tokyo 182-8585, Japan; 13 Faculty of Food and Health Sciences, Showa Women’s University, Tokyo 154-8533, Japan

**Keywords:** subjective well-being, intervention, older adults, isolation, internet

## Abstract

Promoting subjective well-being is a crucial challenge in aging societies. In 2022, we launched a community-based intervention trial (the Chofu-Digital-Choju Movement). This initiative centered on fostering in-person and online social connections to enhance the subjective well-being of older adults. This paper describes the study design and baseline survey. This quasi-experimental study involved community-dwelling older adults aged 65–84 years in Chofu City, Tokyo, Japan. A self-administered questionnaire was distributed to 3742 residents (1681 men and 2061 women), and a baseline survey was conducted in January 2022. We assessed subjective well-being (primary outcome); psychosocial, physical, and dietary factors; and the use of information and communication technology variables (secondary outcomes) among the participants. After the intervention involving online classes, community hubs, and community events, a 2-year follow-up survey will be conducted to evaluate the effects of the intervention, comparing the intervention group (participants) with the control group (non-participants). We received 2503 questionnaires (66.9% response rate); of these, the analysis included 2343 questionnaires (62.6% valid response rate; mean age, 74.4 (standard deviation, 5.4) years; 43.7% male). The mean subjective well-being score was 7.2 (standard deviation, 1.9). This study will contribute to the development of a prototype subjective well-being strategy for older adults.

## 1. Introduction

Japan’s demographic landscape has shown notable progression toward an aging society, with the proportion of the population aged ≥65 years estimated to reach 30% by 2025 [1]. Aging is associated with worsening self-rated health status [2], cognitive impairment [3], and various life events such as bereavement [4,5]. Within this context, subjective well-being is a crucial factor in aging. Well-being is defined as the state where individuals realize their abilities, cope with the normal stresses of life, function productively, contribute to their community, and find contentment [6]. Notably, older adults with high levels of subjective well-being tend to exhibit lower risks of frailty and mortality [7,8,9]. Therefore, it has become imperative to maintain and improve the subjective well-being of older adults in aging societies, aiming to extend their healthy lifespans and overall life expectancy.

Social networks are the determinants of higher subjective well-being. Frequent contact with friends, emotionally positive exchanges, enjoyment, and shared good experiences can contribute to an individual’s subjective well-being [10]. Moreover, psychological intervention programs involving social interactions positively affect subjective well-being [11]. There are four key strategies identified by Suragarn et al. for enhancing social connections among older adults: (1) multi-generational programs, (2) aging-friendly communities, (3) group-based physical activity approaches, and (4) the use of information and communication technology (ICT) [12]. Of these, ICT use warrants particular attention because of its indirect influence on well-being by enhancing social capital [13], which is a feature of social organizations such as networks, norms, and social trust that facilitate coordination and cooperation for mutual benefit [14].

While prior community-based interventions aimed at improving the subjective well-being of older adults have been implemented [15,16,17], none have been community-based with ICT use, to the best of our knowledge. In this context, this study aims to assess the effect of a community-based intervention on improving subjective well-being among the older Japanese population. Accordingly, we launched a study project in 2022 and implemented a multi-component intervention based on strategies to create both in-person and online social connections.

In this study, we describe the study design and baseline characteristics of the participants of the intervention launched in 2022.

## 2. Materials and Method

### 2.1. Study Design, Study Setting, and Participants

The Chofu-Digital-Choju (CDC; Choju means longevity in Japanese) movement is a quasi-experimental study [18] spanning from January 2022 to March 2024, conducted in collaboration with industry, academia, and the government. The CDC movement aims to foster in-person and online social connections to enhance the subjective well-being of older adults. In January 2022, a survey was conducted by mailing a self-administered questionnaire to collect baseline data for the intervention study. A subsequent follow-up survey, following up on the baseline measurements, is scheduled for a 2-year period.

**Figure 1:**
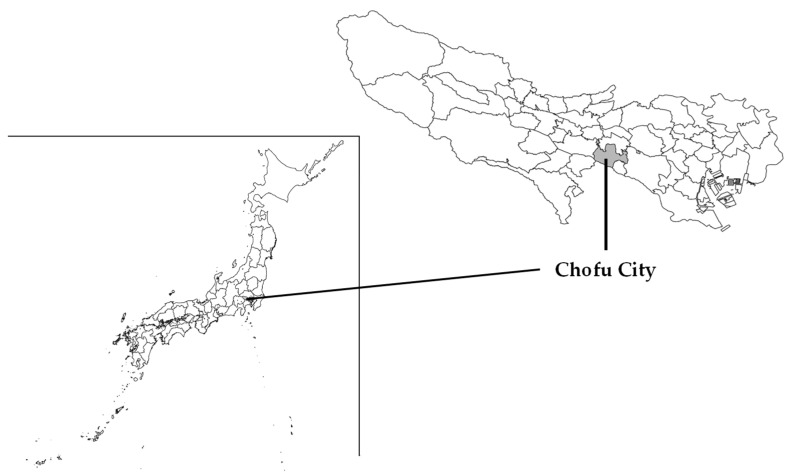
Geographical locations of the study areas (Chofu City, Tokyo, Japan).

The study population comprises community-dwelling individuals aged 65–84 years as of 1 October 2022, living independently in two districts of Chofu City, Tokyo, Japan. Individuals older than 85 years were excluded since the response rate that can be employed was extremely low, according to a previous study [19]. These districts were selected through consultation with city and social welfare council employees based on previous surveys and demographic trends. Chofu City, located in the middle of Tokyo (Figure 1), had a population of 238,311 (115,964 men and 122,347 women) as of 1 October 2021, with 51,536 individuals (22,018 men and 29,518 women) aged ≥65 years, constituting 21.6% of older adults [20]. These districts are located in the south and north of the city, where more than half the households reside in apartment buildings and detached houses, respectively [21].

The sample size was determined through several steps, as follows: first, a previous review by Bolier et al. indicated that positive psychology interventions showed an effect size of 0.34 for subjective well-being [22]. To detect this effect using a *t*-test, 137 individuals were required in both the intervention and control groups (with a statistical power of 0.8 and a risk level of 0.05). Second, based on a previous survey [23], we estimated a dropout rate of approximately 20% over 2 years. Third, the expected valid response rate at baseline and follow-up was approximately 80%, based on previous baseline surveys [19,24]. The percentage of participants required in the intervention group was approximately 10% of the total participants. From these estimates, we calculated a baseline survey sample size of 2675 participants.

**Figure 2:**
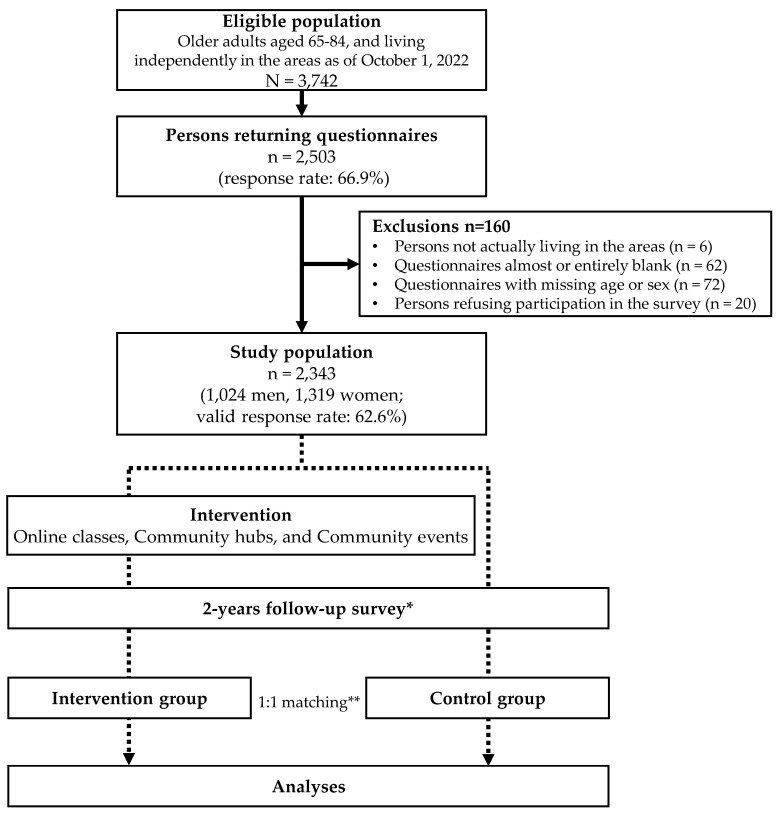
Study flow diagram. * Participants will be categorized into either the intervention or control group based on their response in the follow-up survey regarding prior participation in any of the interventions. ** Propensity score matching with a ratio of 1:1 between the intervention and control groups will be applied using logistic regression based on the baseline survey.

Figure 2 illustrates the study flow diagram. The intervention group consists of participants who received the CDC movement’s intervention, while the control group consists of those who did not. During the 2-year follow-up survey, we will ask the participants whether they engaged in any or all of the interventions, including attending online classes, visiting local community interaction hubs, and joining the community events organized by us. Subsequently, the participants who received at least one of these interventions will be categorized into the intervention group in accordance with the responses in the 2-year follow-up survey.

### 2.2. Baseline and Follow-Up Surveys

As shown in Table 1, the key measurements included subjective well-being as the primary outcome and psychosocial function, physical activity and function, use of ICT, and dietary habits as the secondary outcomes. As additional measures, demographics, socio-economic status, and medical and lifestyle profiles were collected. The self-administered questionnaire, mirroring the items from the baseline survey, will be distributed to the participants, except those who died or relocated from the study area, during the 2-year follow-up survey.

### 2.3. Primary Outcome Measures

Subjective well-being was assessed using the Cantril Ladder, which evaluates people’s attitudes toward their lives and components in various respects [25]. The scale comprised an 11-level Likert scale ranging from 0 (lowest) to 10 (highest).

### 2.4. Secondary Outcome Measures

#### 2.4.1. Psychosocial Function

Social isolation was assessed according to the frequency of outings or contact with family members, relatives, and friends. Social isolation was defined as contact with others less than once a week [26]. Neighborhood relationships were assessed using a four-point scale encompassing “visiting each other”, “standing and chatting”, “exchanging of greetings”, or “none [27,28]”. Additionally, social participation was evaluated based on the frequency of participation in the following activities or groups more than once a month: volunteering, civic action, and nonprofit organizations; sports groups; hobbies and learning groups; senior citizen clubs; neighborhood associations; and others [23].

Psychological aspects include health literacy [29], psychological health [30,31], and depressive mood [32,33].

#### 2.4.2. Physical Activity and Physical Function

Exercise habits were defined as engaging in any exercise one or more times a week and recreational walking or walking for transport for 150 min or more a week [34,35]. Physical function was evaluated according to frailty status [36,37], activities of daily living [38], and the Motor Fitness Scale [39].

#### 2.4.3. Dietary Habits

Dietary variety and food frequency scores were used to evaluate dietary variety [40,41]. The participants were also asked about their status of eating alone [42].

#### 2.4.4. Use of ICT

The questionnaire assessed the type of ICT device used weekly or more frequently and the frequency of Internet usage, such as browsing web pages and exchanging e-mails [43,44].

### 2.5. Intervention Overview

The CDC movement aims to improve the subjective well-being of older adults by creating in-person and online connections. After completing the baseline survey, we will implement the community-based intervention designed by the CDC movement. This intervention consists of online classes, community hubs, and community events.

The online classes integrate an intervention approach combining exercises, nutrition, and cognitive aspects effective in preventing frailty [45]. Additionally, this class employs the Coimagination Method to foster formal conversation techniques aimed at building relationships [46,47]. The exercise component involves workouts designed by professional instructors based on the 10-item muscle training validated in the Japanese Oniishi Model [48,49]. The Oniishi Model’s muscle training is derived from the 19-item regimen by Fiatarone et al. [50] and focuses on fundamental movements such as walking and standing. For safety in an online environment, no specific intensity is designated for the training. The nutrition component, supervised by nutrition experts, includes lectures on diet and food intake for frailty prevention [40]. The lectures center around a mnemonic phrase in Japanese, “sa-a-ni-gi-ya-ka-ni-i-ta-daku” (‘Let’s eat with diversity’), highlighting the importance and techniques of consuming a variety of food groups for frailty prevention [23]. The cognitive aspect involves cognitive enhancement activities led by trainers, such as finger exercises and short-term memory stimulation. The Coimagination Method, designed for equitable participation in communication, comprises two phases, as follows: participants explain their pre-prepared photos and answer questions from others. This process is repeated until all participants have completed both phases. Each session of the online classes focuses on themes such as landscapes, favorite foods, passions, health, and memorable experiences, with facilitation to encourage conversation. Each group, consisting of about ten members, meets once a week for six weeks. The weekly online classes include exercises, nutrition, cognitive aspects, and the Coimagination Method, with participants joining from their homes. Throughout the intervention period, a total of 25 sets of these online classes will be conducted with different participants.

The community hub, serving as a local third place, is operated with the intent of promoting well-being through emotional support provided by volunteers [51,52]. This social interaction center aims to foster trust by listening to and empathizing with the elderly residents of the area. Additionally, it seeks to facilitate social support for older individuals through collaboration with external agencies when necessary [53]. To motivate visits to the hub, health measuring devices are installed, and consultations on smartphone usage are offered. The health measuring devices are capable of assessing or estimating blood pressure, vegetable intake, hemoglobin levels, autonomic nerve function, balance, and cognitive abilities. The hub is open three times a week during the intervention period, with volunteers present during operating hours.

As community events, activities ranging in size from a few to about 30 participants are conducted, being open to all age groups, from youth to the elderly. The themes of these events include general health, exercise, diet, hobbies and entertainment, and the use of ICT such as smartphones. These events are held about four times a month in community centers or university facilities.

### 2.6. Ethical Considerations

The study protocol was approved by the Ethics Committee of the University of Electro-Communications (approval on 16 December 2021; 21068). All the participants provided informed consent. A statement attached to the questionnaire explained the study’s purpose and the voluntary nature of participation and confirmed that the analysis was anonymous. In addition to consent to participate, returning the questionnaire was viewed as consent to participate. All research data are stored on a dedicated computer disconnected from the Internet, and access is exclusively restricted to the members of the research team.

The trial is registered in the UMIN Clinical Trials Registry (UMIN000051393).

### 2.7. Statistical Analyses

All data in the baseline survey are presented as means (standard deviations) or proportions, considering sex differences. The main measures were compared between sexes using the chi-square test for nominal variables and Welch’s *t*-test for continuous variables. A significance level of α = 0.05 indicated statistical significance, and all statistical analyses were performed using IBM SPSS Statistics for Windows, version 29.0 (IBM Corp., Armonk, NY, USA).

As a primary analysis, repeated measures analysis of variance (ANOVA) will be used to verify the interaction between time (pre- and post-intervention) and group (intervention and control groups) to clarify the effect of the interventions on the subjective well-being of older adults after the follow-up survey. In cases where the interaction is significant, a multiple comparison test will be performed using the Bonferroni method. Before ANOVA is performed, propensity score matching with a ratio of 1:1 will be applied using logistic regression based on the baseline survey to reduce bias in background factors between the intervention (participants) and control (non-participants) groups.

A secondary subgroup analysis will be performed to detect differences in the effects of the types of interventions or the intervention frequency on subjective well-being.

## 3. Results

The self-administered questionnaires were distributed to 3742 eligible participants, from whom data were analyzed from 2503 participants (response rate: 66.9%). After excluding 160 questionnaires, including six from respondents living outside the designated areas, 62 questionnaires that were almost entirely blank, 72 that were missing essential identification labels (age or sex), and 20 from respondents who refused to participate in the survey, the analysis included data from 2343 individuals (1024 men and 1319 women; valid response rate: 62.6%).

Table 2 shows the baseline characteristics of the study population. The mean age was 74.4 (standard deviation, 5.4) years. The mean subjective well-being score was 7.2 (standard deviation, 1.9). Compared with women, men were significantly less likely to live alone; have less musculoskeletal pain; have lower subjective well-being, psychological health, activities of daily living, Dietary Variety Score, and Food Frequency Score; and have lower levels of social activities. Additionally, men were significantly more likely to be employed, socially isolated, and frail; have more current drinking and smoking habits; have higher health literacy and Motor Fitness Scale scores; and use the Internet more frequently.

Although physical function deteriorates with aging [54], subjective well-being can be maintained or promoted [22,55,56]. Higher levels of subjective well-being were observed in the participants in this study compared with those in the nationwide survey (the score was 6.2 in 2022) [57]. A previous study revealed that a higher density of older people in certain areas was associated with a higher level of subjective well-being [58]. Although the percentage of the population aged ≥65 years was lower in Chofu City than in Japan as a whole (21.6% vs. 28.9% as of 1 October 2021) [20,59], the participants had higher subjective well-being.

## 4. Discussion

To the best of our knowledge, the present study describes a unique intervention because it facilitates the formation of online and in-person communities; provides group-based online programs consisting of physical exercise, cognitive training, and nutrition lectures; establishes community hubs with psychological support from volunteers; and organizes multi-generational community events. These attempts to create connections for older adults may extend their healthy life expectancy, mediated by maintaining or promoting subjective well-being.

Previous reviews have indicated that ICT use can reduce loneliness and social isolation [60]; however, older individuals who use the Internet have a higher socioeconomic status than non-users [61]. In this baseline survey, the proportion of older adults using the Internet was lower than that in a national survey in Japan (60.1%) [62]. The promotion of Internet and ICT use through this intervention can allow community-dwelling older adults to obtain its benefits, leading to reduced social isolation.

This study has some limitations. First, the participation rate was not high compared to that in some cohort studies in the baseline survey [19,24,63]. Second, the low proportion of participants using the Internet, compared with a national survey in Japan, can potentially influence the results. Third, the study’s internal validity may have been affected by participants’ recall bias, as a self-administered questionnaire was used. Fourth, although the random selection of participants and the calculation of sample size for interventions would typically be performed, the present study was conducted as a quasi-experimental study. Finally, in this community-based intervention, there is a potential risk of contamination that could affect the accurate assessment of intervention effects, as both the intervention and control groups reside in the same area. Therefore, the fact that all participants belong to the same residential area may affect the results of the study.

The CDC movement was launched in 2022 to create connections to enhance subjective well-being among community-dwelling older adults. A multi-component intervention and the planned 2-year follow-up survey will be implemented. This study is expected to contribute to the development of a prototype strategy to promote the subjective well-being of older adults.

## 5. Conclusions

The community-based intervention trial (the Chofu-Digital-Choju movement) was launched in 2022 to improve the subjective well-being of community-dwelling older adults by fostering in-person and online connections. The insights of this study could contribute to the development of a prototype subjective well-being strategy for older adults.

## Figures and Tables

**Table 1:** Summary of the items surveyed at baseline, 2022.

**Primary outcome measures**
Subjective well-being
**Secondary outcome measures**
Psychosocial function
Social isolation
Neighborhood relationships
Social participation
Health literacy (CCHL)
Psychological health (WHO-5)
Depressive mood (GDS-5)
Physical activity and physical function
Exercise habits
Frailty status (CL15)
Activities of daily living (TMIG-IC)
Motor Fitness Scale
Dietary habits
Dietary Variety Score
Food Frequency Score
Eating alone
Use of ICT
Type of own ICT
Frequency of Internet usage
**Additional measures**
Age
Sex
Cohabiters
Years of residence in the neighborhood area
Financial status
Employment status
Chronic musculoskeletal pain (shoulder, low back, knee)
Body mass index (self-rated height and weight)
Drinking and smoking

CCHL: communicative and critical health literacy; WHO: World Health Organization; GDS-5: 5-item version of the Geriatric Depression Scale; CL15: Check-List 15; TMIG-IC: Tokyo Metropolitan Institute of Gerontology Index of Competence; ICT: information and communication technology.

**Table 2:** Baseline characteristics of the participants.

Variables	All		Men		Women		*p*-Value
Number of residents aged 65–84 years old	3742		1681		2061		
Number of analyzed participants (eligible response rate; %)	2343	(62.6)	1024	(60.9)	1319	(64.0)	
Age, years, mean (SD)	74.4	(5.4)	74.2	(5.5)	74.5	(5.3)	0.094
65–74, *n* (%)	1232	(52.6)	564	(55.1)	668	(50.6)	
75–84, *n* (%)	1111	(47.4)	460	(44.9)	651	(49.4)	0.033
Living alone, *n* (%)	454	(19.5)	157	(15.4)	297	(22.7)	<0.001
Year of residence in the neighborhood, *n* (%)							
<29	718	(30.8)	337	(33.0)	381	(29.0)	
30–59	1407	(60.3)	561	(54.9)	846	(64.4)	
>59	209	(9.0)	123	(12.0)	86	(6.5)	<0.001
Financial status (self-rated), *n* (%)							
Low	76	(3.3)	42	(4.1)	34	(2.6)	
Middle-low	303	(13.0)	143	(14.1)	160	(12.2)	
Middle	888	(38.2)	361	(35.5)	527	(40.3)	
Middle-high	941	(40.5)	419	(41.2)	522	(39.9)	
High	118	(5.1)	52	(5.1)	66	(5.0)	0.055
Employment status, *n* (%)							
No	1537	(66.5)	581	(57.0)	956	(73.9)	
Yes	776	(33.5)	439	(43.0)	337	(26.1)	<0.001
Musculoskeletal pain (either shoulder, low back, or knee), *n* (%)							
No	792	(34.8)	391	(39.1)	401	(31.5)	
Yes	1482	(65.2)	610	(60.9)	872	(68.5)	<0.001
Body mass index, kg/m^2^, mean (SD)	22.7	(3.2)	23.3	(2.9)	22.2	(3.3)	<0.001
<18.5, *n* (%)	178	(7.8)	38	(3.8)	140	(10.9)	
18.5–24.9, *n* (%)	1632	(71.1)	719	(71.4)	913	(70.9)	
≥25, *n* (%)	485	(21.1)	250	(24.8)	235	(18.2)	<0.001
Alcohol drinking status, *n* (%)							
Never or former	1026	(44.0)	274	(26.8)	752	(57.4)	
Current	1307	(56.0)	749	(73.2)	558	(42.6)	<0.001
Smoking status, *n* (%)							
Never or former	2116	(90.8)	865	(84.7)	1251	(95.5)	
Current	215	(9.2)	156	(15.3)	59	(4.5)	<0.001
**Primary outcome measures**							
Subjective well-being, mean (SD)	7.2	(1.9)	6.9	(1.9)	7.4	(1.8)	<0.001
**Secondary outcome measures**							
Social relationships							
Social isolation, *n* (%)	1572	(68.6)	782	(78.1)	790	(61.2)	<0.001
Neighborhood relationships, *n* (%)							
Visiting each other	489	(21.8)	132	(13.3)	357	(28.4)	
Standing and chatting	921	(41.0)	344	(34.6)	577	(46.0)	
Exchanging of greetings	694	(30.9)	421	(42.4)	273	(21.8)	
None	144	(6.4)	96	(9.7)	48	(3.8)	<0.001
Social participation more than once a month, *n* (%)	1032	(44.6)	346	(34.1)	686	(52.9)	<0.001
Health literacy (CCHL), mean (SD)	3.6	(0.8)	3.6	(0.8)	3.5	(0.8)	0.028
Psychological health (WHO-5: 0–25), mean (SD)	15.0	(5.4)	14.6	(5.6)	15.3	(5.3)	0.004
Depressive mood (GDS-5 ≥2), *n* (%)	814	(36.0)	365	(36.8)	449	(35.5)	0.526
Physical activity and physical function							
Engaging in any exercise more than once a week, *n* (%)	1735	(76.8)	756	(76.1)	979	(77.4)	0.456
Engaging in walking 150 or more minutes per week, *n* (%)	1473	(65.0)	615	(62.1)	858	(67.2)	0.012
Going out more than once a day, *n* (%)	1504	(65.1)	672	(66.7)	832	(63.8)	0.136
Frailty (CL15 score ≥4), *n* (%)	497	(22.8)	252	(26.4)	245	(20.0)	<0.001
TMIG-IC (score: 0–13), mean (SD)	11.3	(1.8)	10.9	(2.1)	11.6	(1.6)	<0.001
Motor Fitness Scale, mean (SD)	10.5	(3.5)	10.9	(3.2)	10.1	(3.7)	<0.001
Dietary variety							
Dietary Variety Score (0–10), mean (SD)	3.4	(2.3)	2.8	(2.2)	3.9	(2.3)	<0.001
Food Frequency Score (0–30), mean (SD)	18.4	(5.2)	16.9	(5.1)	19.6	(4.9)	<0.001
Eat alone at least a whole day per week, *n* (%)	1041	(46.6)	442	(44.7)	599	(48.1)	0.112
Use of ICT							
Owing smartphone	1529	(66.2)	677	(67.0)	852	(65.6)	0.488
Using the Internet more than once a day, *n* (%)	1059	(46.7)	545	(54.6)	514	(40.5)	<0.001

CCHL: communicative and critical health literacy; WHO: World Health Organization; GDS-5: 5-item version of the Geriatric Depression Scale; CL15: Check-List 15; TMIG-IC: Tokyo Metropolitan Institute of Gerontology Index of Competence; ICT: information and communication technology; SD: standard deviation. Missing values were eliminated, and *p*-values were calculated using the chi-square test for categorical variables, and Welch’s *t*-test was employed to compare continuous variables between men and women.

## Data Availability

The data presented in this study are available on request from the corresponding author.

## References

[1] Cabinet Office Japan Annual Report on the Ageing Society FY2022 (Entire Edition). https://www8.cao.go.jp/kourei/whitepaper/w-2022/html/zenbun/index.html.

[2] Andersen F.K., Christensen K., Frederiksen H. (2007). Self-rated health and age: A cross-sectional and longitudinal study of 11,000 Danes aged 45–102. Scand. J. Public. Health.

[3] Matsui Y., Tanizaki Y., Arima H., Yonemoto K., Doi Y., Ninomiya T., Sasaki K., Iida M., Iwaki T., Kanba S. (2009). Incidence and survival of dementia in a general population of Japanese elderly: The Hisayama study. J. Neurol. Neurosurg. Psychiatry.

[4] Hughes M.E., Waite L.J. (2009). Marital biography and health at mid-life. J. Health Soc. Behav..

[5] Laditka J.N., Laditka S.B. (2003). Increased hospitalization risk for recently widowed older women and protective effects of social contacts. J. Women Aging.

[6] World Health Organization Mental Health Action Plan 2013–2020. https://www.who.int/publications-detail-redirect/9789241506021.

[7] Gale C.R., Cooper C., Deary I.J., Aihie Sayer A. (2014). Psychological well-being and incident frailty in men and women: The English longitudinal study of ageing. Psychol. Med..

[8] Chida Y., Steptoe A. (2008). Positive psychological well-being and mortality: A quantitative review of prospective observational studies. Psychosom. Med..

[9] Minagawa Y., Saito Y. (2023). Subjective well-being and active life expectancy in Japan: Evidence from a longitudinal Study. Innov. Aging.

[10] Pinquart M., Sörensen S. (2000). Influences of socioeconomic status, social network, and competence on subjective well-being in later life: A meta-analysis. Psychol. Aging.

[11] Iwano S., Kambara K., Aoki S. (2022). Psychological interventions for well-being in healthy older adults: Systematic review and meta-analysis. J. Happiness Stud..

[12] Suragarn U., Hain D., Pfaff G. (2021). Approaches to enhance social connection in older adults: An integrative review of literature. Aging Health Res..

[13] Kokubun K., Ogawa T., Browne R., Shinada T., Granrath L., Moeller J., Tram N., Wieching R., Taki Y. (2022). Social capital mediates the association between the ICT usage and well-being of older People in Japan: Implication for a new design paradigm. Sustainability.

[14] Putnam R.D., Alone B., Reader A., Crothers L., Lockhart C. (2000). America’s declining social capital. Culture and Politics.

[15] Gasteiger C., Collens P., du Preez E. (2023). Community-based support to improve mental health and wellbeing in older sexually and gender diverse people: A scoping review. Aging Ment. Health.

[16] Foettinger L., Albrecht B.M., Altgeld T., Gansefort D., Recke C., Stalling I., Bammann K. (2022). The Role of community-based men’s sheds in health promotion for older men: A mixed-methods systematic review. Am. J. Mens. Health.

[17] Giebel C., Shrestha N., Reilly S., White R.G., Zuluaga M.I., Saldarriaga G., Liu G., Allen D., Gabbay M. (2022). Community-based mental health and well-being interventions for older adults in low- and middle-income countries: A systematic review and meta-analysis. BMC Geriatr..

[18] Handley M.A., Lyles C.R., McCulloch C., Cattamanchi A. (2018). Selecting and improving quasi-experimental designs in effectiveness and implementation research. Annu. Rev. Public. Health.

[19] Yamashita M., Seino S., Nofuji Y., Sugawara Y., Osuka Y., Kitamura A., Shinkai S. (2022). The Kesennuma Study in Miyagi, Japan: Study design and baseline profiles of participants. J. Epidemiol..

[20] (2021). Chofu City Households and Population in Chofu City. https://www.city.chofu.tokyo.jp/www/contents/1610018912361/index.html.

[21] Statistics of Japan Population Census 2020. https://www.e-stat.go.jp/stat-search/files?page=1&layout=datalist&toukei=00200521&tstat=000001136464&cycle=0&tclass1=000001136472&tclass2=000001159886&cycle_facet=tclass1%3Acycle&tclass3val=0.

[22] Bolier L., Haverman M., Westerhof G.J., Riper H., Smit F., Bohlmeijer E. (2013). Positive psychology interventions: A meta-analysis of randomized controlled studies. BMC Public. Health.

[23] Seino S., Tomine Y., Nishi M., Hata T., Fujiwara Y., Shinkai S., Kitamura A. (2021). Effectiveness of a community-wide intervention for population-level frailty and functional health in older adults: A 2-year cluster nonrandomized controlled trial. Prev. Med..

[24] Seino S., Kitamura A., Tomine Y., Tanaka I., Nishi M., Nonaka K., Nofuji Y., Narita M., Taniguchi Y., Yokoyama Y. (2019). A community-wide intervention trial for preventing and reducing frailty among older adults living in metropolitan areas: Design and baseline survey for a study integrating participatory action research with a cluster trial. J. Epidemiol..

[25] Glatzer W., Gulyas J., Michalos A.C. (2014). Cantril self-anchoring striving scale. Encyclopedia of Quality of Life and Well-Being Research.

[26] Saito M., Kondo K., Ojima T., Hirai H., JAGES group (2015). Criteria for social isolation based on associations with health indicators among older people a 10-year follow-up of the Aichi gerontological evaluation study. Nihon Koshu Eisei Zasshi.

[27] Seino S., Kitamura A., Nishi M., Tomine Y., Tanaka I., Taniguchi Y., Yokoyama Y., Amano H., Narita M., Ikeuchi T. (2018). Individual- and community-level neighbor relationships and physical activity among Older Japanese adults living in a metropolitan area: A cross-sectional multilevel analysis. Int. J. Behav. Nutr. Phys. Act..

[28] Nakamura H., Nakamura M., Okada E., Ojima T., Kondo K. (2017). Association of food access and neighbor relationships with diet and underweight among community-dwelling Older Japanese. J. Epidemiol..

[29] Ishikawa H., Nomura K., Sato M., Yano E. (2008). Developing a measure of communicative and critical health literacy: A pilot study of Japanese office workers. Health Promot. Int..

[30] Awata S., Bech P., Yoshida S., Hirai M., Suzuki S., Yamashita M., Ohara A., Hinokio Y., Matsuoka H., Oka Y. (2007). Reliability and validity of the Japanese version of the World Health Organization-five well-being index in the context of detecting depression in diabetic patients. Psychiatry Clin. Neurosci..

[31] Awata S., Bech P., Koizumi Y., Seki T., Kuriyama S., Hozawa A., Ohmori K., Nakaya N., Matsuoka H., Tsuji I. (2007). Validity and utility of the Japanese version of the WHO-five well-being index in the context of detecting suicidal ideation in elderly community residents. Int. Psychogeriatr..

[32] Hoyl M.T., Alessi C.A., Harker J.O., Josephson K.R., Pietruszka F.M., Koelfgen M., Mervis J.R., Fitten L.J., Rubenstein L.Z. (1999). Development and testing of a five-item version of the Geriatric Depression Scale. J. Am. Geriatr. Soc..

[33] Rinaldi P., Mecocci P., Benedetti C., Ercolani S., Bregnocchi M., Menculini G., Catani M., Senin U., Cherubini A. (2003). Validation of the five-item Geriatric Depression Scale in elderly subjects in three different settings. J. Am. Geriatr. Soc..

[34] Kamada M., Kitayuguchi J., Inoue S., Ishikawa Y., Nishiuchi H., Okada S., Harada K., Kamioka H., Shiwaku K. (2013). A community-wide campaign to promote physical activity in middle-aged and elderly People: A cluster randomized controlled trial. Int. J. Behav. Nutr. Phys. Act..

[35] Nelson M.E., Rejeski W.J., Blair S.N., Duncan P.W., Judge J.O., King A.C., Macera C.A., Castaneda-Sceppa C. (2007). Physical activity and public health in older adults: Recommendation from the American College of Sports Medicine and the American Heart Association. Med. Sci. Sports Exerc..

[36] Shinkai S., Watanabe N., Yoshida H., Fujiwara Y., Amano H., Lee S., Nishi M., Tsuchiya Y. (2010). Research on Screening for Frailty: Development of “the Kaigo-Yobo Checklist”. Nihon Koshu Eisei Zasshi.

[37] Shinkai S., Watanabe N., Yoshida H., Fujiwara Y., Nishi M., Fukaya T., Lee S., Kim M.J., Ogawa K., Murayama H. (2013). Validity of the “Kaigo-Yobo Check-List” as a frailty index. Nihon Koshu Eisei Zasshi.

[38] Koyano W., Shibata H., Nakazato K., Haga H., Suyama Y. (1991). Measurement of competence: Reliability and validity of the TMIG index of competence. Arch. Gerontol. Geriatr..

[39] Kinugasa T., Nagasaki H. (1998). Reliability and validity of the motor fitness scale for older adults in the community. Aging.

[40] Kumagai S., Watanabe S., Shibata H., Amano H., Fujiwara Y., Shinkai S., Yoshida H., Suzuki T., Yukawa H., Yasumura S. (2003). Effects of dietary variety on declines in high-level functional capacity in elderly people living in a community. Nihon Koshu Eisei Zasshi.

[41] Kimura M., Moriyasu A., Kumagai S., Furuna T., Akita S., Kimura S., Suzuki T. (2013). Community-based intervention to improve dietary habits and promote physical activity among older adults: A cluster randomized trial. BMC Geriatr..

[42] Tani Y., Sasaki Y., Haseda M., Kondo K., Kondo N. (2015). Eating Alone and depression in older men and women by cohabitation status: The JAGES longitudinal survey. Age Ageing.

[43] LaMonica H.M., Davenport T.A., Roberts A.E., Hickie I.B. (2021). Understanding technology preferences and requirements for health information technologies designed to improve and maintain the mental health and well-being of older adults: Participatory design Study. JMIR Aging.

[44] Duplaga M. (2021). The association between Internet use and health-related outcomes in older adults and the elderly: A cross-sectional study. BMC Med. Inform. Decis. Mak..

[45] Ng T.P., Feng L., Nyunt M.S.Z., Feng L., Niti M., Tan B.Y., Chan G., Khoo S.A., Chan S.M., Yap P. (2015). Nutritional, physical, cognitive, and combination interventions and frailty reversal among older adults: A randomized controlled trial. Am. J. Med..

[46] Otake M., Kato M., Takagi T., Asama H. (2011). The Coimagination method and its evaluation via the conversation interactivity measuring method. Early Detection and Rehabilitation Technologies for Dementia: Neuroscience and Biomedical Applications.

[47] Mihoko O.-M. (2018). Conversation assistive technology for maintaining cognitive health. J. Korean Gerontol. Nurs..

[48] Asakawa Y., Endo F., Yamaguchi H., Takahashi R. (2008). The characteristics of participants and the effects of self-paced resistance training for community-dwelling elders in early period of a residents-led preventive care program, the Onishi Model. Rigaku Ryōhōgaku.

[49] Matsubayashi Y., Asakawa Y., Yamaguchi H. (2016). Low-frequency group exercise improved the motor functions of community-dwelling elderly People in a rural area when combined with home exercise with self-monitoring. J. Phys. Ther. Sci..

[50] Fiatarone M.A., Marks E.C., Ryan N.D., Meredith C.N., Lipsitz L.A., Evans W.J. (1990). High-intensity strength training in nonagenarians. Effects on skeletal muscle. JAMA.

[51] Marzuki A.A., Nor N.N.F.M., Rashid S.M.R.A., Ghazali S. (2023). Social support by communities for older adults in Malaysia. Nurture.

[52] Choi E., Han K.M., Chang J., Lee Y.J., Choi K.W., Han C., Ham B.J. (2021). Social participation and depressive symptoms in community-dwelling older adults: Emotional social support as a mediator. J. Psychiatr. Res..

[53] Colistra C., Bixler R., Schmalz D. (2019). Exploring factors that contribute to relationship building in a community center. J. Leis. Res..

[54] Collard R.M., Boter H., Schoevers R.A., Oude Voshaar R.C. (2012). Prevalence of frailty in community-dwelling older persons: A systematic review. J. Am. Geriatr. Soc..

[55] Tiefenbach T., Kohlbacher F., Eckermann E. (2014). Subjective well-being across gender and age in Japan: An econometric analysis. Gender, Lifespan and Quality of Life: An International Perspective.

[56] Blanchflower D.G., Oswald A.J. (2008). Is well-being U-shaped over the life cycle?. Soc. Sci. Med..

[57] Helliwell J.F., Layard R., Sachs J.D., Neve J.E.D., Aknin L.B., Wang S. (2023). World Happiness Report.

[58] Saito T., Sugisawa H., Harada K., Kai I. (2016). Population aging in local areas and subjective well-being of older adults: Findings from two studies in Japan. BioSci Trends.

[59] Statistics Bureau of Japan Current Population Estimates as of 2021. https://www.stat.go.jp/english/data/jinsui/2021np/index.html.

[60] Döring N., Conde M., Brandenburg K., Broll W., Gross H.M., Werner S., Raake A. (2022). Can communication technologies reduce loneliness and social isolation in older People? A scoping review of reviews. Int. J. Environ. Res. Public. Health.

[61] Hunsaker A., Hargittai E. (2018). A review of Internet use among older adults. New Media Soc..

[62] (2022). Ministry of Internal Affairs and Communications the Communications Usage Trend Survey. https://www.soumu.go.jp/johotsusintokei/statistics/statistics05.html.

[63] Murayama H., Nofuji Y., Matsuo E., Nishi M., Taniguchi Y., Fujiwara Y., Shinkai S. (2014). The Yabu cohort Study: Design and profile of participants at baseline. J. Epidemiol..

